# The *C. elegans* SET1 histone methyltransferase SET-2 is not required for transgenerational memory of silencing

**DOI:** 10.17912/micropub.biology.001143

**Published:** 2024-05-13

**Authors:** Cécile Bedet, Piergiuseppe Quarato, Francesca Palladino, Germano Cecere, Valérie J Robert

**Affiliations:** 1 Ecole Normale Supérieure de Lyon, Laboratory of Biology and Modeling of the Cell, CNRS UMR5239, Inserm U1293, University Claude Bernard Lyon 1, 69007 Lyon, France Auvergne-Rhône-Alpes, France; 2 Mechanisms of Epigenetic Inheritance, Department of Developmental and Stem Cell Biology, Institut Pasteur, CNRS UMR3738, Paris, France; 3 Current address: San Raffaele Telethon Institute for Gene Therapy, IRCCS San Raffaele Scientific Institute, Milan, Italy

## Abstract

The SET-2 /SET1 histone H3K4 methyltransferase and RNAi pathway components are required to maintain fertility across generations in *C. elegans* . SET-2 preserves the germline transcriptional program transgenerationally, and RNAi pathways rely on small RNAs to establish and maintain transgenerational gene silencing. We investigated whether the functionality of RNAi-induced transgenerational silencing and the composition of pools of endogenous small RNA are affected by the absence of SET-2 . Our results suggest that defects in RNAi pathways are not responsible for the transcriptional misregulation observed in the absence of SET-2 .

**Figure 1. The transgenerational memory of silencing and small RNA pools are intact in the absence of SET-2:**
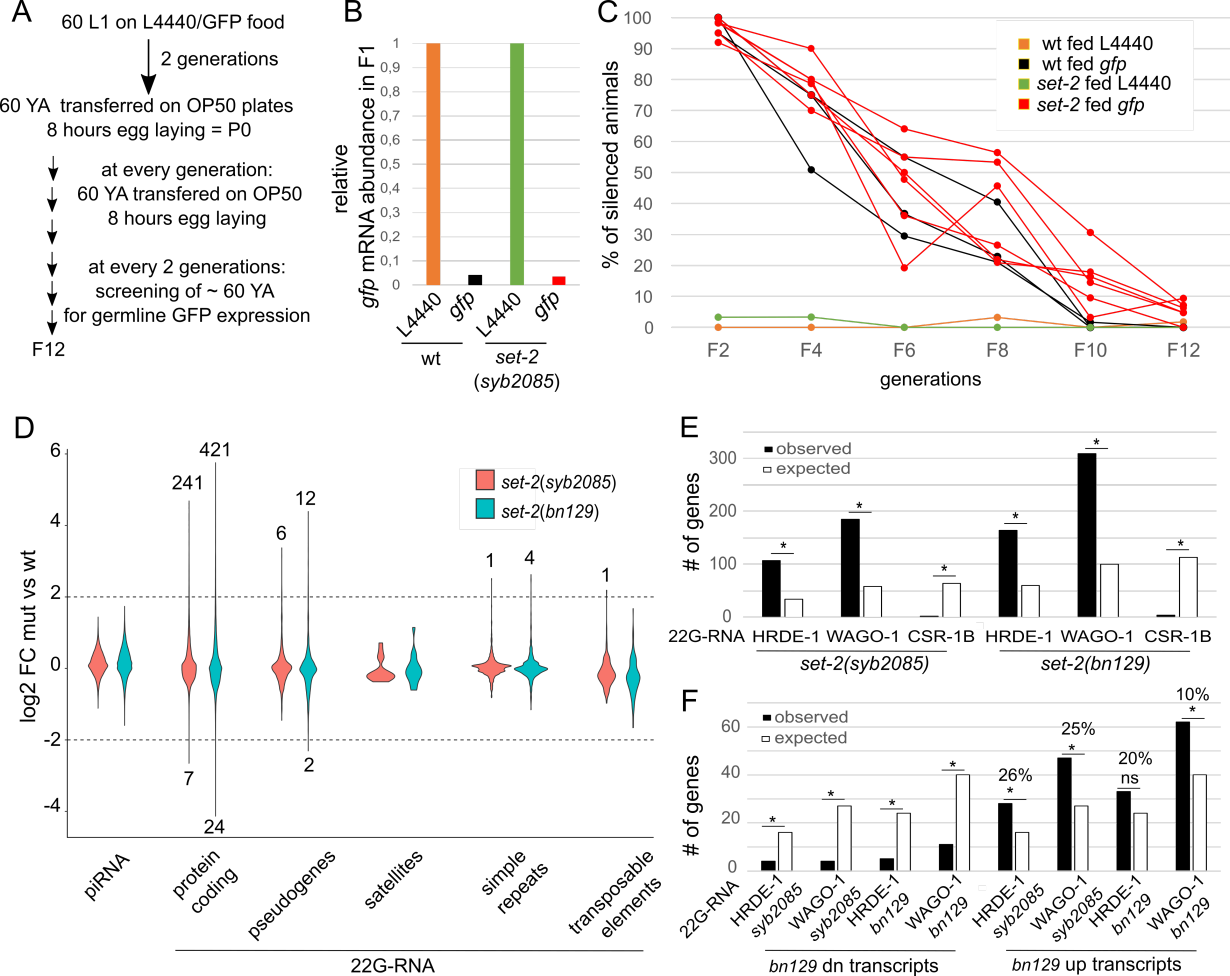
(A) Experimental design of RNAi induction and monitoring of transgenerational memory of silencing. (B) qRT-PCR quantification of *gfp* mRNA abundance in F1 wild-type and *set-2* ( *syb2085* ) animals treated for 2 generations with L4440 (empty vector) or dsRNA GFP food. Color code is the same as in (C). (C) Transgenerational scoring of silenced wild-type and *set-2* ( *syb2085* ) animals fed with L4440 or dsRNA GFP food. 3 independent wild-type and 6 *set-2* ( *syb2085* ) lines were analyzed. (D) Small RNA pool sequencing in *set-2(bn129)* and *set-2(syb2085)* animals. Depleted and enriched piRNA and 22G-RNA (classified according to the nature of their genomic targets). Number of targets with significantly misregulated small RNA (log2FC(mut vs wt) < -2 and log2FC(mut vs wt) > 2) are indicated. (E) Classification of *set-2* enriched 22G-RNA according to their association with the 3 Argonaute proteins HRDE-1, WAGO-1 and CSR-1B in wild-type animals. Expected numbers and statistical significance were calculated using a hypergeometric assay. (F) Comparison of enriched HRDE-1 and WAGO-1 22G-RNA with genes misregulated in *set-2* ( *bn129* ) germlines. Percent of genes enriched with HRDE-1- or WAGO-1- associated 22G-RNA in *set-2* mutants and upregulated in *set-2* ( *bn129* ) germlines are indicated. Expected numbers and statistical significance were calculated using a hypergeometric assay. The list of misregulated genes in *set-2* ( *bn129* ) mutant germlines is from (Herbette *et al.* 2020).

## Description

In *C. elegans* , transgenerational loss of fertility, also referred to as the mortal germline (Mrt) phenotype, is observed in both wild isolates and mutant strains grown in laboratory conditions (Ahmed and Hodgkin 2000; Smelick and Ahmed 2005; Frézal et al. 2018; 2023). Mutants with mortal germlines fall into two classes: those that become sterile at all temperatures, and those that display a reversible temperature-sensitive Mrt (tsMrt) phenotype at the non-permissive temperature (25°C). Mutants in the first class identified genes involved in telomere replication, genome stability and histone methylation (Ahmed and Hodgkin 2000; Meier et al. 2006, 2009; Andersen and Horvitz 2007; Katz et al. 2009) . Mutants in the second class define RNAi pathway components and chromatin associated proteins including SET-2 , the *C. elegans* homolog of the SET1 H3K4 histone methyltransferase that plays context dependent roles in transcription (Simonet et al. 2007; Li and Kelly 2011; Xiao et al. 2011; Buckley et al. 2012; Sakaguchi et al. 2014; Spracklin et al. 2017; Howe et al. 2017; Weiser et al. 2017; Saltzman et al. 2018; Manage et al. 2020; Wan et al. 2021; Caron et al. 2021; Seroussi et al. 2023) .

RNAi pathways are key players in the regulation of gene expression both at the transcriptional and post-transcriptional levels (Billi et al. 2014; Almeida et al. 2019; Seroussi et al. 2022) . They rely on primary RNA signals that trigger RdRP-dependent production of secondary 22G-RNAs. 22G-RNAs are amplified through a self-sustaining loop, and can be transmitted to the progeny for several generations without the primary trigger, thereby playing an essential role in the transgenerational inheritance of gene expression regulation (Gu et al. 2012) . It has been proposed that alteration in the composition of 22G-RNA pools can result in the deregulation of transcriptional programs over generations, ultimately leading to loss of sterility (Buckley et al. 2012) . Supporting this model, it was shown that the absence of piRNAs (one type of primary RNA signal triggering RNAi pathways) affects the pools of 22G-RNA targeting histone mRNAs over generations. As a result, histone mRNAs are silenced and animals become sterile (Barucci et al. 2020) .

We and others have previously reported that the tsMrt phenotype observed in the absence of SET-2 correlates with loss of germline identity and transgenerational deregulation of the germline transcriptional program (Xiao et al. 2011; Robert et al. 2014, 2020; Caron et al. 2021) . In this study, we asked if transgenerational sterility and progressive deregulation of transcription programs observed in the absence of SET-2 result from a functional defect in RNAi pathways. In wild-type animals, single-copy transgenes expressing a Green Fluorescent Protein (GFP) under the control of germline promoters can be silenced when animals are fed dsRNA molecules targeting *GFP* , and this silencing is transmitted over 9 to 12 generations after elimination of the dsRNA trigger (Buckley et al. 2012) . To test whether this silencing process is still functional in the absence of SET-2 , we fed *set-2 ( syb2085 )* animals carrying a catalytically dead allele of *set-2* and the *oxIs279* [ *pie-1p* :: *gfp* ::H2B + *unc-119* (+)] transgene expressed in the germline with a bacteria clone expressing *gfp* dsRNA ( Figure 1A ) (Frøkjær-Jensen et al. 2008; Caron et al. 2021). We then monitored GFP silencing and re-expression across generations after the elimination of the dsRNA trigger. Both wild-type and *set-2* ( *syb2085 )* animals show a substantial decrease in mRNA *gfp,* which correlates with efficient silencing of the *oxIs279* transgene observed by fluorescent imaging ( Figure 1B and 1C). Following removal of dsRNA, 100% of the wild-type animals re-expressed GFP at generation F10 in 3 independent lines, while 9-30% of the *set-2* ( *syb2085* ) animals at the same generation still showed GFP silencing in 5 out of 6 independent lines. By the F12 generation, 4-9% of the *set-2* ( *syb2085* ) animals were still silenced in 5 out of 6 independent lines. Statistical analysis (chi-square test, see Methods section) performed on pooled data at F10 and F12 shows that the small difference observed between wild-type and mutant animals is significant, suggesting that RNAi silencing memory induced at the *oxIs279* transgene may be slightly longer in the absence of SET-2 .

Next, we asked whether *set-2* mutant display misregulation of small RNA pools that could correlate with altered germline transcriptional programs and result in transgenerational loss of fertility. We sequenced the small RNA pools present in *set-2* ( *syb2085 )* animals *,* also including *set-2* ( *bn129 )* animals carrying a null allele of *set-2* in our analysis. While similar pools of piRNA were found in *set-2* mutants and wild-type animals, we identified 249 and 437 DNA elements (including protein coding genes, pseudogenes and repetitive elements) with significantly more 22G-RNAs (mean log2 foldchange ( *set-2* vs WT) > 2) in *syb2085* and *bn129* mutants respectively than in wild-type. A few DNA elements with significantly less 22G-RNAs in *syb2085* and *bn129* mutants than in wild-type ( Figure 1D ) were also found. We further analyzed 241 and 421 genes with more 22G-RNAs in *syb2085* and *bn129* mutants, respectively, and that target protein coding genes. We found that, in wild-type animals, this set of genes is enriched for targets of HRDE-1- and WAGO-1-associated 22G-RNAs, and depleted for targets of CSR-1-associated 22G-RNAs ( Figure 1E ). In wild-type germlines, 22G-RNA associated with HRDE-1 and WAGO-1 are involved in germline gene silencing (Gu et al. 2009; Buckley et al. 2012) . By contrast, in *set-2* mutant germlines we observed that instead of correlating with downregulation of target genes, overproduction of this subset of HRDE-1- or WAGO-1 associated 22G-RNA correlates with upregulation of target genes with 12% to 26% of the genes enriched for HRDE-1- or WAGO-1-associated-22G-RNA in *set-2* mutants being upregulated in *set-2* ( *bn129* ) mutant germlines ( Figure 1F ). We speculate that the overproduction of 22G-RNAs in this context may result from increased transcription resulting in greater availability of mRNA template. At this stage, it is not possible to conclude whether overproduced 22G-RNA have any regulatory function on gene expression. However, their presence might disturb the balance between small RNA pathways and account for the slightly longer transgenerational memory that we observed in the absence of SET-2 .

In conclusion, SET-2 does not significantly contribute to the mechanisms underlying small RNA pathways, and small RNA pathway deregulation is unlikely to be responsible for the transgenerational loss of fertility in *set-2* mutants. This conclusion fits with our previous observation that SET-2 works in parallel with the NRDE pathway (involved in the heritability of RNAi silencing of gene expression) to support germline immortality (Robert et al. 2014) .

## Methods

dsRNA induced GFP silencing and monitoring of GFP re-expression

For RNAi induction of silencing, L4440 and dsRNA GFP clones were grown overnight at 37°C in liquid LB media complemented with Ampicillin. dsRNA expression was induced by adding IPTG to the liquid culture to a final concentration of 1mM and bacteria were grown at 37°C for 2 additional hours before seeding 1 ml on standard NGM plates complemented with 1 mM IPTG. PFR733 and PFR725 strains were synchronized by sodium hypochlorite treatment and 60 L1 animals of each strain were transferred on RNAi (L4440 and dsRNA GFP) plates. Animals developed for 48 hours at 20°C and were transferred as young adults on fresh RNAi plates for a second generation of silencing induction. Their progeny (considered as P0 in our experiment) were transferred in pools of 60 animals on OP50 plates and allowed to lay eggs for 8 hours before elimination. This “transfer/egg laying” procedure was repeated every 3 days and GFP re-expression was scored every 2 generations on 50-60 young adults immobilized on agarose pad using an AZ100M Nikon macrozoom microscope. Animals were scored as “re-expressing” GFP as soon a signal could be detected. A chi-square test was perfomed at the F10 and F12 generations on pooled data (F10, WT: 1 GFP- animal and 191 GFP+ animals, *set-2* ( *syb2085* ) : 61 GFP- animals and 317 GFP+ animals. Chi2 = 30.3 p-value = 3.66x10 ^-8^ ; F12, WT = 0 GFP- and 112 GFP+, *set-2* ( *syb2085* ) : 8 GFP- and 119 GFP+ ; chi2 = 7.29 and p-value = 0.007).

qRT-PCR

Mix stage populations were harvested at F1 generation, washed in M9 buffer and resuspended in 300 µl of NucleoZOL (Macherey-Nagel #740404-200). RNA was extracted according to manufacturer's protocol using Nucleospin RNA Set for NucleoZOL kit (Macherey-Nagel #740406.50). RNA was eluted in 40 µL of UltraPure water and the integrity and concentration of RNA was measured with TapeStation 4200 and RNA Screen Tape (Agilent). RNAs were retrotranscribed using the Transcriptor Universal cDNA Master kit (Roche #05893151001). qPCRs were performed with Takyon SYBR 2X MasterMix (Eurogentec #UF-NSMT-B0705) on a CFX real-time detection system (CFX 96 Biorad) and *gfp* RNA levels were normalized to the mean of *act-1* and *cdc-42* genes. primers: act-1 _for: gctggacgtgatcttactgattacc / act-1 _rev: gtagcagagcttctccttgatgtc / cdc-42 _for: ctgctggacaggaagattacg / cdc-42 _rev: ctcggacattctcgaatgaag / gfp_for: ggcggtaccggtagaaaaa / gfp_rev: ttgtgcccattaacatcacc.

small RNA sequencing and analysis

For small RNA sequencing, worm sorting was performed using a COPAS Biosorter (Union Biometrica) to obtain a population of worms enriched for the young adult stage (Cornes et al. 2022) . The mutant worms were collected four generations after homozygosis to obtain enough worms for sorting. Total RNA with RIN > 9 was used to generate small RNA libraries. The library preparation and data analysis were performed as previously described (Barucci et al. 2020) .

## Reagents

**Table:** 

strain	genotype	from
N2	wildtype	sunybiotech
EG4601	*oxIs279* [ *Ppie-1::GFP::H2B* + *unc-119* (+)] II, *unc-119* ( *ed3* ) III	CGC
PHX2171	*set-2* ( *syb2085* ) III/ *qC1* [ *dpy-19* ( *e1259* ) *glp-1* ( *q339* ) *qIs26* ] III	(Caron et al. 2021)
PFR733	*oxIs279* ( *Ppie-1::GFP::H2B* ) II	This study
PFR725	*set-2* ( *syb2085* ) III; *oxIs279* ( *Ppie-1::GFP::H2B* ) II	This study
PFR510	*set-2 ( bn129 ) III/ qC1 [ dpy-19 ( e1259 ) glp-1 ( q339 ) qIs26 ] III *	(Robert et al. 2020)

**Table:** 

plasmid	information	from
GFP feeding clone	Full-length GFP cDNA cloned between 2 T7 promoters to produce GFP dsRNA.	Gift from Marie-Anne Félix.
L4440 feeding clone	Empty vector	(Fire et al. 1998)
